# Association of diabetes, hypertension and obesity with cognitive impairment in adults

**DOI:** 10.1590/1980-5764-DN-2025-0471

**Published:** 2026-08-24

**Authors:** Sara Beatriz Raimann, Nathalia Avila Dimer, Bárbara Niegia Garcia de Goulart

**Affiliations:** 1 Universidade Federal do Rio Grande do Sul, Porto Alegre RS, Brazil.; 2 Universidade Federal do Rio Grande do Sul, Instituto de Psicologia, Serviço Social, Saúde e Comunicação Humana, Porto Alegre RS, Brazil.

**Keywords:** Metabolic Diseases, Cognition, Aging, Multimorbidity., Doenças Metabólicas, Cognição, Envelhecimento, Multimorbidade.

## Abstract

Cognitive impairment (CI) is prevalent in aging and affects the functionality and autonomy of individuals. Previous studies have demonstrated its association with metabolic diseases such as diabetes *mellitus* (DM), systemic arterial hypertension (SAH), hypercholesterolemia, and obesity. Still, the synergic association of these comorbidities in the Brazilian population has not yet been explored. Elucidating the magnitude of the relationship between metabolic diseases and CI informs the planning of more effective health care and attention strategies.

**Objective:**

To investigate the association between DM, SAH, hypercholesterolemia and obesity and CI in adults aged 50 years or older.

**Methods:**

Cross-sectional population-based study using data from the second wave of the Brazilian longitudinal study of aging — ELSI-Brazil (2019). A total of 6,449 participants with complete data on the variables of interest and who responded directly to the survey were included in the analysis. CI was defined using standardized test z-scores. Exposures were identified through self-reported medical diagnoses and waist circumference measurements.

**Results:**

The combination of DM, hypertension, and obesity was associated with a 4% increase in the prevalence of CI. With the addition of hypercholesterolemia, this increase reached 10%. The isolated or combined presence of only two of these conditions was not associated with significant differences.

**Conclusion:**

Among people aged 50 and over, CI is more prevalent in those with multiple metabolic alterations. In a scenario of aging and high burden of chronic diseases, the findings reinforce the urgency of integrated prevention and control strategies to contain CI.

## RESUMO

O déficit cognitivo (DC) é prevalente no envelhecimento e afeta a funcionalidade e a autonomia dos indivíduos. Estudos têm demonstrado sua associação com doenças metabólicas, como diabetes *mellitus* (DM), hipertensão arterial sistêmica (HAS), hipercolesterolemia e obesidade. No entanto, na população brasileira a ação sinérgica dessas doenças ainda não foi explorada. Elucidar a magnitude da relação entre essas doenças metabólicas associadas e o DC contribui para planejar estratégias de cuidado e atenção em saúde mais eficazes.

**Objetivo:**

Investigar a associação entre DM, HAS, hipercolesterolemia e obesidade e o DC em adultos com 50 anos ou mais.

**Métodos:**

Estudo transversal de base populacional, com dados da segunda onda do Estudo Longitudinal da Saúde dos Idosos Brasileiros — ELSI-Brasil (2019). Foram incluídos 6.449 participantes com dados completos das variáveis de interesse e que responderam diretamente à pesquisa. O DC foi definido com base no escore z de testes padronizados. As exposições foram identificadas por meio de autorrelato de diagnóstico médico e da medida da circunferência da cintura.

**Resultados:**

A combinação de DM, HAS e obesidade associou-se a um aumento de 4% na prevalência de DC. Com a adição da hipercolesterolemia, esse aumento atingiu 10%. A presença isolada ou combinada de apenas duas dessas condições não se associou a diferenças significativas.

**Conclusão:**

Entre pessoas com 50 anos ou mais, o DC é mais prevalente entre as que apresentam múltiplas alterações metabólicas. Em um cenário de população longeva e alta prevalência de idosos com DCNT, os achados reforçam a urgência de estratégias integradas de prevenção e identificação precoce para a oferta de tratamento oportuno para casos de DC.

## INTRODUCTION

Globally, the population over 60 years of age has increased significantly. Estimates from the World Health Organization (WHO) suggest that, by 2050, there will be approximately 2.1 billion people aged 60 or older worldwide^1^. Brazil is following the same path; from 2010 to 2022, the number of individuals over 65 years of age rose from 14.1 million to 22.2 million, representing 11% of the country’s population^2^.

In this context of population aging, multimorbidity — characterized by the co-occurrence of two or more diseases—is frequently observed in the elderly^3^. Systemic arterial hypertension (SAH), diabetes mellitus (DM), hypercholesterolemia, and obesity are among the most common conditions. Data published in 2024 indicate that these metabolic diseases are those that most affect and cause harm throughout life, not only in the elderly but also in adults aged 50 and older^4^. Their co-occurrence is explained by insulin resistance, and their combined action synergistically affects endothelial dysfunction, although not predictably. Metabolic changes should therefore be examined using additive and multiplicative models^5 6^.

These conditions are also established modifiable risk factors for other outcomes, such as dementia, and represent a substantial burden on healthcare systems^7^. A systematic review^8^ conducted in 2009 compared the individual effects of DM, SAH, dyslipidemia, and obesity on cognition in older adults, finding consistent associations for DM and SAH, while results for dyslipidemia and obesity were less conclusive. More recently, reports from the Lancet Commission on Dementia included obesity in 2017^9^ and LDL cholesterol in 2024^10^ as modifiable risk factors. It is estimated that up to 12% of dementia cases worldwide could be prevented or reduced if DM, SAH, obesity, and high LDL cholesterol were eliminated^10^.

Dementia is characterized by impairment in two or more cognitive functions, along with a decline in daily activities, whereas mild cognitive impairment (MCI) compromises only one cognitive function. Both, however, negatively impact individuals’ ability to function independently in activities of daily living and social aspects. The WHO estimates that, by 2050, more than 152 million people will be affected by dementia, and suggests that it is strongly associated with aging. The WHO also emphasizes that dementia should not be considered natural and expected, and can be prevented, as shown by the aforementioned studies^11^.

The relationship between each of the metabolic alterations in isolation and cognition is widely studied; however, to date, we have not found studies that concomitantly consider DM, SAH, hypercholesterolemia and obesity and their association with cognitive impairment (CI) in the Brazilian population, given its specific characteristics^12^. Since all these conditions greatly compromise the functionality, autonomy and well-being of affected individuals and their support and care network^13^, in addition to generating high costs for health systems^14^, the objective of this study is to investigate the association between DM, SAH, hypercholesterolemia and obesity and CI in Brazilian adults aged 50 years or older.

## METHODS

### Study design

This cross-sectional study used data from the second wave of ELSI-BRASIL, a national longitudinal survey conducted in 70 Brazilian municipalities with populations aged 50 years and older. The selection and stratification of research areas were based on the geographic operational database of the Brazilian Institute of Geography and Statistics (IBGE). Municipalities were stratified by population size, followed by a selection of census tracts and eligible households for interviews and physical measurements.

The second wave was conducted from 2019 to 2021, coordinated by the Federal University of Minas Gerais (UFMG) in partnership with the Oswaldo Cruz Foundation, Minas Gerais (FIOCRUZ-MG), and funded by the Ministry of Health. The present study used public, anonymized data and, according to resolution 466/12 of the Brazilian National Health Council (CNS), did not require approval from a Research Ethics Committee^15^.

### Population and sample

From 9,949 participants in wave 2 of ELSI-Brazil (2019–2021), those with two waist circumference measurements and information on physician diagnosis of hypertension, diabetes, and high cholesterol were included. Exclusions comprised 1,992 without both waist measures, 72 without hypertension data, 104 without diabetes data, and 175 without cholesterol data.

**Figure 1:**
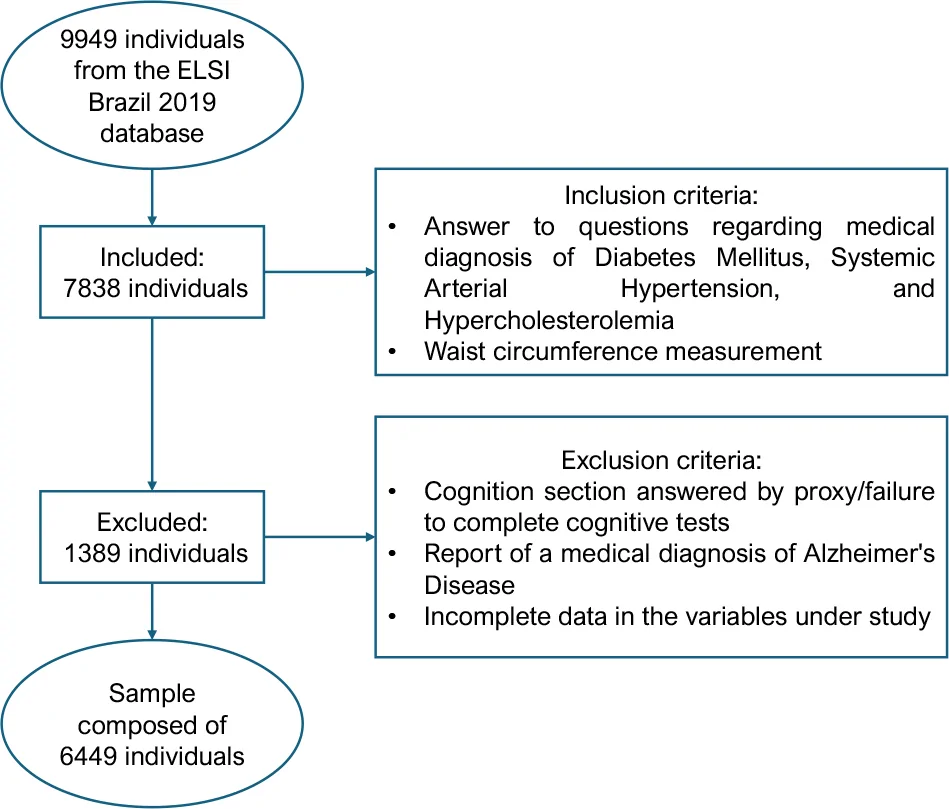
Sample composition

Source: Elaborated by the authors.

Participants with proxy responses for cognition, without language, memory, or temporal orientation tests, with self-reported Alzheimer’s disease (question n63), or missing adjustment variables were also excluded. The final sample included 6,449 participants. The sample composition process is detailed in Figure 1.

### Outcome

ELSI-Brazil cognitive tests are comparable to the Health and Retirement Study and the Mexican Health and Aging Study, enabling international comparisons^15^. Global cognition included language, memory, and temporal orientation.

Language was assessed by naming a fruit and an object from descriptions, and identifying the Brazilian president and vice-president at the time. Temporal orientation included day, month, year, and weekday, recorded and classified as correct or incorrect. Memory was assessed by immediate and delayed recall of ten words.

Z-scores were calculated for each domain as (observed value minus mean) divided by standard deviation (SD). Memory included immediate and delayed recall scores. Language and temporal orientation assigned 1 point per correct answer, totaling 4 points per domain. Global cognition was the mean of domain scores, standardized as a z-score. Cognitive impairment (CI) was defined as z-score ≤-1 SD.

### Exposure

DM, SAH, and hypercholesterolemia were obtained by self-report of prior medical diagnosis. Original categories were recoded as “yes” or “no”, considering pregnancy-related diagnosis as “no”. Individuals with “don’t know” or missing responses were excluded.

Obesity was defined by mean waist circumference^16^ from two measurements (question mf17m). The inelastic Seca tape was positioned midway between the 10^th^ rib and the edge of the iliac crest. Measurements were taken at eye level and directly on the participant’s skin. The participant was standing with bare feet apart, abdomen relaxed, and breathing normally. Participants were considered obese when waist circumference was equal or greater than 88 cm for women and 102 cm for men^17^.

From these data, new dichotomous variables (categorized as “yes” or “no”) were created, considering: individuals with two or more metabolic alterations and individuals with all four studied alterations. Also for possible harmonizations of the concomitance of three of the studied alterations, resulting in four new variables: individuals with DM, SAH, and hypercholesterolemia; individuals with DM, SAH, and obesity; individuals with DM, hypercholesterolemia, and obesity; and individuals with SAH, hypercholesterolemia, and obesity. New variables were also created for the possible combinations of two of the studied alterations: DM and SAH, DM and hypercholesterol; DM and obesity; SAH and hypercholesterolemia; SAH and obesity; and hypercholesterolemia and obesity^6^.

### Data analysis

Data are presented by absolute and relative frequencies, considering sample weights and presented to the second decimal place, rounded up when the third decimal place is ≥5. Poisson regression with robust variance was used to calculate prevalence estimates and 95% confidence intervals (95%CI) of the dependent variable “cognitive impairment” (overall), dichotomized into “yes” and “no”, for each of the independent variables. The reference category for the independent variables in all analyses was “no”, grouping individuals who did not have the set of diseases under analysis. For adjustment, the sociodemographic variables sex (male and female), age (50≤59, 60≤69, 70 or older), self-reported race (White, Black/mixed race, others) and education (up to four years of study and five years of study or more) were used, as well as the variable physical activity practice (practice was considered when activities of moderate or intense intensity were performed, three or more times a week)^18^.

## RESULTS

Among the participants aged 50 years or older of this study, 55.1% were female, 55.3% self-identified as Black or Mixed Race, and 15.6% lived in rural areas. The mean age was 62.5 years (SD ±8.9), and 49.5% of respondents reported having no or up to four years of formal education.

The prevalence of CI among the study participants was 13.5%. Of the study participants, 75.9% had some self-reported metabolic disease, 47% had two or more concomitantly, and 4.4% had all four self-reported metabolic diseases: DM, SAH, hypercholesterolemia, and obesity. Participant characteristics according to the CI outcome are presented in Table 1.

**Table 1:** Sociodemographic and lifestyle characterization of study participants, cognitive impairment.

	Cognitive impairment		Total
	Yes	No	100%*
	13.5%*	86.5%*	
Age (years)			
50–59	30.3	52.1	49.2
60–69	29.6	29.3	29.3
70 or older	40.1	18.6	21.5
Sex			
Female	63.6	53.8	55.1
Male	36.4	46.2	44.9
Residence area			
Rural	25.4	14.0	15.6
Urban	74.6	86.0	84.4
Race			
White	44.1	44.1	44.1
Black/Brown	55.5	55.3	55.3
Another	0.4	0.5	0.5
Education (years of study)			
5 or more	20.2	55.2	50.5
<5	79.8	44.8	49.5
Metabolic diseases			
0	19.8	24.8	24.1
1	31.6	30.9	31
≥2	48.7	44.3	47
Practice of physical activity			
Yes	28.1	40.4	38.7
No	71.9	59.6	61.3

Note: *Weighted values.

Source: Elaborated by the authors.

In the crude regression analysis, individuals with two or more metabolic alterations had a prevalence ratio (PR) of 1.16 (95%CI 1.16–1.16), representing a 16% higher chance of CI when compared to their peers with one or no metabolic alteration. In the analysis adjusted for the sociodemographic characteristics model, individuals with two or more metabolic alterations had a PR of 0.93 (95%CI 0.93–0.93) of presenting CI when compared to their peers, reducing to PR of 0.91 (95%CI 0.91–0.91) in the final model (model of sociodemographic characteristics plus the physical activity variable), suggesting that the crude association can be explained by the adjusted characteristics: sex, age, education, and physical activity.

When analyzing the possible associations between two of the metabolic diseases studied and their crude association with global CI, we found for those with DM and SAH a PR of 1.28 (95%CI 1.27–1.28), for DM and hypercholesterolemia PR 1.08 (95%CI 1.07–1.08), DM and obesity PR 1.17 (95%CI 1.16–1.17), SAH and hypercholesterolemia PR 1.21 (95%CI 1.20–1.21), SAH and obesity PR 1.25 (95%CI 1.24–1.25), and hypercholesterolemia and obesity PR 1.11 (95%CI 1.10–1.11). Concomitant DM and SAH demonstrated, after adjustments for sociodemographic and lifestyle factors, PR 1.02 (95%CI 1.01–1.02), DM and hypercholesterolemia PR 0.88 (95%CI 0.88–0.89), DM and obesity PR 0.98 (95%CI 0.97–0.98), SAH and hypercholesterolemia PR 0.96 (95%CI 0.96–0.97), SAH and obesity PR 0.95 (95%CI 0.95–0.95), and hypercholesterolemia and obesity PR 0.91 (95%CI 0.91–0.92) of association with global CI when compared to their peers.

In individuals with three metabolic alterations, the crude association with CI demonstrates a variation between the different sets of metabolic alterations: having DM, SAH and hypercholesterolemia demonstrated PR 1.20 (95%CI 1.20–1.20), having DM, SAH and obesity presented PR 1.35 (95%CI 1.35–1.35), the concomitant presence of DM, hypercholesterolemia and obesity demonstrated PR 1.25 (95%CI 1.25–1.26) and having SAH, hypercholesterolemia and obesity resulted in PR 1.29 (95%CI 1.29–1.30). After adjustments, having DM, SAH and hypercholesterolemia obtained PR 0.95 (95%CI 0.94–0.95), having DM, SAH and obesity demonstrated PR 1.04 (95%CI 1.03–1.04), the concomitant presence of DM, hypercholesterolemia and obesity demonstrated PR 0.99 (95%CI 0.99–1.00), and having SAH, hypercholesterolemia and obesity resulted in PR 1.00 (95%CI 0.99–1.00) of association with global CI when compared to their peers.

The association between global CI and concomitant self-reported DM, SAH and hypercholesterolemia and abdominal obesity showed, in the crude estimate, a PR of 1.46 (95%CI 1.46–1.47); adjusted by the final model, considering sociodemographic and lifestyle variables, the estimated PR was 1.10 (95%CI 1.10–1.11), demonstrating a 10% higher prevalence of association with global CI when compared to pairs with three or fewer metabolic alterations. The crude and adjusted results of the analyses of the association between CI and all possible sets of two and three, as well as the four metabolic diseases investigated, are presented in Table 2.

**Table 2:** Association between metabolic alterations and global cognitive impairment in adults aged 50 years or older in Brazil (2019).

	Crude PR	p-value	Model 1	p-value	Model 2	p-value	Model 3	p-value
	PR (95%CI)		PR (95%CI)		PR (95%CI)		PR (95%CI)	
Having two or more diseases	1.16 (1.16–1.16)	0.000	0.93 (0.93–0.93)	0.000	1.12 (1.12–1.12)	0.000	0.91 (0.91–0.91)	0.000
DM x SAH	1.28 (1.27–1.28)	0.000	1.03 (1.03–1.04)	0.000	1.23 (1.23–1.23)	0.000	1.02 (1.01–1.02)	0.000
DM x HC	1.08 (1.07–1.08)	0.000	0.91 (0.90–0.91)	0.000	1.04 (1.04–1.04)	0.000	0.88 (0.88–0.89)	0.000
DM x Obesity	1.17 (1.16–1.17)	0.000	1.00 (1.00–1.01)	0.018	1.11 (1.10–1.11)	0.000	0.98 (0.97–0.98)	0.000
SAH x HC	1.21 (1.20–1.21)	0.000	0.98 (0.98–0.98)	0.000	1.18 (1.17–1.18)	0.000	0.96 (0.96–0.97)	0.000
SAH x Obesity	1.25 (1.24–1.25)	0.000	0.97 (0.96–0.97)	0.000	1.20 (1.20–1.20)	0.000	0.95 (0.95–0.95)	0.000
HC x Obesity	1.11 (1.10–1.11)	0.000	0.93 (0.93–0.93)	0.000	1.07 (1.07–1.07)	0.000	0.91 (0.91–0.92)	0.000
DM x SAH x HC	1.20 (1.20–1.20	0.000	0.97 (0.96–0.97)	0.000	1.16 (1.15–1.16)	0.000	0.95 (0.94–0.95)	0.000
DM x SAH x Obesity	1.35 (1.35–1.35)	0.000	1.06 (1.06–1.07)	0.000	1.28 (1.28–1.28)	0.000	1.04 (1.03–1.04)	0.000
DM x HC x Obesity	1.25 (1.25–1.26)	0.000	1.02 (1.02–1.03)	0.000	1.18 (1.18–1.19)	0.000	0.99 (0.99–1.00)	0.000
SAH x HC x Obesity	1.29 (1.29–1.30)	0.000	1.02 (1.02–1.02)	0.000	1.25 (1.24–1.25)	0.000	1.00 (0.99–1.00)	0.180
DM x SAH x HC x Obesity	1.46 (1.46–1.47)	0.000	1.14 (1.13–1.14)	0.000	1.38 (1.38–1.39)	0.000	1.10 (1.10–1.11)	0.000

Abbreviations: PR, prevalence ratio; CI, confidence interval; DM, diabetes mellitus; SAH, sistemic arterial hypertension; HC, hypercholesterolemia.

Source: Elaborated by the authors.

## DISCUSSION

Among Brazilian adults aged 50 years or older participating in this study, the prevalence of CI was 13.5%. In the crude analyses, all results were significant, with associations between 8 and 46% higher between the possible combinations of the self-reported metabolic diseases analyzed and CI compared to their peers. When the associations between the combinations of self-reported metabolic diseases and CI were adjusted for sex, age, education, race, and physical activity, it was observed that concomitant self-reported DM, SAH, and obesity increased the prevalence of CI by 4% (PR 1.04; 95%CI 1.03–1.04), and self-reported DM, SAH, obesity, and hypercholesterolemia increased it by 10% (PR 1.10; 95%CI 1.10–1.11). The remaining combinations of self-reported metabolic diseases were not statistically significantly associated with CI after adjustment. It should be noted that women appear to be at higher risk of developing CI, while older age and lower education are known risk factors for CI. Studies have shown that regular physical activity can prevent the development of CI and dementia. These factors, all characteristic of the research sample, may account for the associations observed in the crude models.

Studies investigating the associations between concomitant metabolic and cardiac diseases and CI^19,20,21^ report that individuals with multiple metabolic and/or cardiac diseases have lower scores on cognitive tests compared to their peers. These score differences, however, are not always significant, and there is no agreement between studies regarding the effect of the diseases studied on cognitive functioning. Variations across studies may be due to population differences (social, environmental, genetic) and measurement tools.

When analyzing the specific combinations between two or three of the diseases studied, the associations after adjusting for sex, age, education, race, and physical activity practice became insignificant for the most part, with only the combination of DM, SAH, and obesity demonstrating a 4% (PR 1.04; 95%CI 1.03–1.04) greater association with CI when compared to their peers. A study that investigated metabolic syndrome — a group of at least three of five metabolic alterations, including those we investigated—and its relationship with CI^22^ found stronger associations. Still, we consider that these differences are due to the more severe disease state, in addition to analyzing the specific effects on cognitive domains such as processing speed and attention, for example.

In those with all four metabolic alterations concomitantly, the crude analysis resulted in a 46% association, and after adjustments, a 10% greater association with CI was observed among these individuals compared with those with three or fewer alterations. This result, as we initially hypothesized, demonstrates that an increased number of metabolic diseases is associated with a higher prevalence of CI. A study conducted with four cohorts comparable to ELSI-Brazil — Health and Retirement Study, English Longitudinal Study of Ageing, the Survey of Health, Ageing and Retirement in Europe, and China Health and Retirement Longitudinal Study^23^ — investigated the longitudinal association of the cardiometabolic diseases DM, heart disease, and stroke with CI. They found that individuals with at least one of these diseases were more likely also to have SAH and obesity, and those with a greater number of cardiometabolic diseases showed greater decline in cognitive function over time (one cardiometabolic disease β -0.02; 95%CI -0.04–0.01, two cardiometabolic diseases β -0.13; 95%CI -0.17 to -0.09, and three cardiometabolic diseases β -0.22; 95%CI -0.29 to -0.14). Although this is a more severe multimorbidity profile, the results of that study reinforce a decline in cognitive function among individuals aged 50 years and older with cardiometabolic multimorbidity.

Some limitations should be clarified: first, we were unable to control participants’ medication use, as well as the time elapsed from the diagnosis of the diseases to participation in the research, which may interfere by diminishing the effects of disease on the body^24 25^. There is a possibility of recall bias in verifying medical diagnoses through self-reporting. Because this was a cross-sectional study, cause-and-effect relationships could not be estimated, nor could we determine the time elapsed between the diagnosis of metabolic alterations and the outcome. Longitudinal research is needed to elucidate the causal mechanism underlying the associations described.

Nevertheless, a large and representative sample of the Brazilian population aged 50 and over strengthens the applicability of our results to this population, as well as the standardization of cognitive tests and their administration by trained interviewers. This is the first study that aims to investigate the effect of self-reported DM, SAH, hypercholesterolemia, and obesity combined, and their association with CI in people aged 50 years or older in the Brazilian population, a developing country in Latin America. The study presents prevalence rates for having two or more of these diseases, as well as for each of the possible combinations in which individuals may be affected by two, three, or all four of the metabolic diseases studied.

In conclusion, in this study, the prevalence of CI in individuals aged 50 or older living in Brazil was 13.5%. The concomitant presence of self-reported DM, SAH, and obesity was associated with a 4% increase in the prevalence of CI compared to peers without this combination of diseases. When self-reported hypercholesterolemia was added to this condition, the prevalence increased to 10%, suggesting a synergistic effect between metabolic diseases on CI. Notably, hypercholesterolemia alone did not show a significant association in the other analyses, supporting the hypothesis that interactions among multiple metabolic conditions may potentiate the risk of CI.

The findings highlight the clinical and epidemiological relevance of the association between multiple metabolic diseases and CI in adults aged 50 years and older. In a context of population aging and a high incidence of chronic diseases, with direct consequences for the social and professional functioning of these individuals, health systems face increasing challenges due to the demand for diagnostic, monitoring, and specialized support services.

Given the lack of pharmacological or interventional therapies capable of reversing or halting CI, investment in preventive and multidisciplinary strategies is essential, with a focus on early identification of risk factors and on promoting metabolic health throughout life. The intersection between epidemiology, clinical practice, and public policy must be strengthened to mitigate the impact of CI on the aging population.

## Data Availability

The datasets generated and analyzed during the current study are available at ELSI Brasil Study official website (https://elsi.cpqrr.fiocruz.br/data-access/) upon request.
